# The context of biotechnology - The biotechnologuy sector in Brazil. Going up?

**DOI:** 10.1186/1753-6561-8-S4-O47

**Published:** 2014-10-01

**Authors:** Eduardo Emrich

**Affiliations:** 1 Biominas, Minas Gerais, Brazil

## 

The presentation will discuss the status of the biotechnology sector in Brazil.

We see a lot of attention in the last months to the Health Ministry effort to decrease the health trade deficit by establishing technology transfer and production agreements evolving international and local companies and public institutions. The emergence of companies as Orygen and Bionovis and the announced projects conducted by Biomm and Recepta are part of this strategy.

On the other hand, we also see a complex scenario for start-ups in the bio field. In fact, it is even more difficult for entrepreneurs or researchers to start a company in Brazil due to the lack of programs to support them.

On the second stage, start-ups have to deal with an unbelievable regulatory environment where companies have to wait two or more years to start operations. And no financing: non-refundable resources are scarce at FINEP, the brazilian innovation agency. It is also hard for a start-up to find investors, as there are few funds investing in life sciences projects. So, companies are financed by a combination of research/scientific programs, state level programs and, in some cases, angel investors.

Management is also a trouble for the start-ups. Only a few have dedicated business teams, scientific interaction is small and collaborations with local or international companies are rare. Whether these are causes of consequences of the financial situation has still to be determined.

Also, the environment for biotechnology has to be analyzed. We see a lack of crucial components in the product development chain in the country, from pre-clinical labs, translational centers to biologics manufacturing facilities. There are not many professional business incubators and technology parks too. And also, it is not easy to find trained people in product development.

In the short term, the development of the bioindustry in Brazil will depend on how these big projects will perform. In case of success, they could create a positive cycle, encouraging new projects and creating a positive legal and regulatory environment. Will all the other companies have to wait ? Or can we use different strategies together ?

